# Novel immortalized human fetal liver cell line, cBAL111, has the potential to differentiate into functional hepatocytes

**DOI:** 10.1186/1472-6750-9-89

**Published:** 2009-10-21

**Authors:** Tanja Deurholt, Niek P van Til, Aniska A Chhatta, Lysbeth ten Bloemendaal, Ruth Schwartlander, Catherine Payne, John N Plevris, Igor M Sauer, Robert AFM Chamuleau, Ronald PJ Oude Elferink, Jurgen Seppen, Ruurdtje Hoekstra

**Affiliations:** 1 AMC Liver Center, Meibergdreef 69-71, 1105 BK Amsterdam, the Netherlands; 2 Department. of Surgery (Surgical Laboratory), Academic Medical Center, Meibergdreef 9, 1105 AZ Amsterdam, the Netherlands; 3 Experimental Surgery and Regenerative Medicine, Charité, Augustenburger Platz 1, 13353 Berlin, Germany; 4 Department of Hepatology, Royal Infirmary of Edinburgh, 49 Little France crescent, Edinburgh EH16 4SB, UK

## Abstract

**Background:**

A clonal cell line that combines both stable hepatic function and proliferation capacity is desirable for *in vitro *applications that depend on hepatic function, such as pharmacological or toxicological assays and bioartificial liver systems. Here we describe the generation and characterization of a clonal human cell line for *in vitro *hepatocyte applications.

**Results:**

Cell clones derived from human fetal liver cells were immortalized by over-expression of telomerase reverse transcriptase. The resulting cell line, cBAL111, displayed hepatic functionality similar to the parental cells prior to immortalization, and did not grow in soft agar. Cell line cBAL111 expressed markers of immature hepatocytes, like glutathione S transferase and cytokeratin 19, as well as progenitor cell marker CD146 and was negative for lidocaine elimination. On the other hand, the cBAL111 cells produced urea, albumin and cytokeratin 18 and eliminated galactose. In contrast to hepatic cell lines NKNT-3 and HepG2, all hepatic functions were expressed in cBAL111, although there was considerable variation in their levels compared with primary mature hepatocytes. When transplanted in the spleen of immunodeficient mice, cBAL111 engrafted into the liver and partly differentiated into hepatocytes showing expression of human albumin and carbamoylphosphate synthetase without signs of cell fusion.

**Conclusion:**

This novel liver cell line has the potential to differentiate into mature hepatocytes to be used for *in vitro *hepatocyte applications.

## Background

Most pharmacological or toxicological assays and bioartificial liver support systems require fully differentiated hepatocytes. The availability of mature human hepatocytes is variable and the numbers low, because they are usually isolated from donor livers not suitable for transplantation. In addition these cells hardly proliferate *in vitro *[1,2]. Since mature human hepatocytes cannot be used for large-scale applications, there is a pressing need for a cell line that combines highly differentiated hepatic functions while maintaining adequate proliferation capacity.

Several cell lines derived from human liver tumours, such as the hepatoma cell line HepG2 [3], as well as *in vitro *immortalized cell lines, like the NKNT-3 cell line, have been investigated [4,5]. In general, these cell lines proliferate adequately, but the levels of hepatocyte-specific functions (*e.g*. urea production from ammonia and cytochrome p450 detoxification activity) remain disappointedly low.

In tumour-derived cell lines, the mutations leading to immortalization are largely unknown. In an attempt to control the immortalization process and therefore prevent at least part of the dedifferentiation process, several immortalized cell lines have been developed. However, although certain genetic modifications in immortalized cell lines are known, spontaneous mutations contributing to the immortalization cannot be excluded.

For successful *in vitro *immortalization, overexpression of cell cycle stimulating genes is generally required. Due to the low proliferation capacity of mature hepatocytes, strong stimulation of cell cycle progression is necessary for immortalization. In the majority of *in vitro *immortalizations of primary human liver cells, the gene encoding Simian Virus 40 Large T antigen (SV40T), an inhibitor of the cell cycle inhibitors p53 and the Retinoblastoma protein, has been used [4,6-8]. In addition, overexpression of Cyclin D1, which stimulates cell cycle progression, and dominant negative mutants of p53 have also led to successful immortalization [9]. In some immortalized cell lines, proliferation was combined with stabilisation of the telomeres [10]. Critically short telomeres induce a terminal state of growth arrest called crisis [11]. Overexpression of the reverse transcriptase of telomerase, hTERT, stabilizes telomere length, thereby avoiding cellular crisis. As a general principle immortalization by overexpression of hTERT only, minimises the reduction in functionality [12].

In contrast to mature human hepatocytes, fetal human hepatocytes have the ability to proliferate *in vitro *[13,12] and thereby can be immortalized without cell cycle stimulation. In addition, telomere stabilisation in itself can immortalize these cells [12]. Wege *et al *showed that the immortalization of fetal human hepatocytes did not affect their differentiation potential, however, the functionality of the immortalized cells was not compared with mature human hepatocytes. Such comparison is essential to establish the suitability of these cells for hepatocyte applications *in vitro*. Furthermore phenotypical stability of these cells may be low, since these were not of clonal origin. In our previous report we demonstrated that primary human fetal liver cells (HFLCs) in culture exhibit albumin production rates and hepatocyte specific mRNA levels comparable to those of primary mature human hepatocytes *in vitro *[14]. However, after eight population doublings most of these functions were decreased to less than 1% of the corresponding function of primary human hepatocytes *in vitro*. This functional loss can be, at least partly, attributed to the presence of non-parenchymal cells in the cell preparation, which eventually outnumber the functional hepatocytes. Therefore, selection of functional cell clones is necessary if HFLCs are extensively expanded *in vitro*.

In a previous study we already isolated HFLCs and selected specific clones based on their morphology and growth potential [14]. This study combines the telomerase based immortalization technique with the selection of functional HFLCs to obtain new hepatic cell lines. The resulting immortalized cell line was tested for hepatic functionality *in vitro *and *in vivo*. The *in vitro *functionality was compared with other well-known hepatic cell lines, more specifically the conditionally immortalized NKNT-3 [4] and the tumour derived HepG2 cells [3]. To test whether the resulted novel immortalized cell line had the ability to differentiate into functional hepatocytes, the cell line was transplanted into the spleen of immunodeficient mice.

## Methods

### Cell isolation and culture

Human fetal livers were obtained from elective abortions. Gestational age was determined by ultrasonic measurement of the diameter of the skull and ranged from 14 to 18 weeks. The use of this tissue was approved by the Medical Ethical Committee of the Academic Medical Center, Amsterdam, the Netherlands, subject to informed patient consent in compliance with the Helsinki Declaration. We isolated HFLCs on three independent occasions; in each case four fetal livers were pooled. Cells were isolated as described previously [14]. HFLCs were seeded in DMEM culture medium (Dulbecco's modified Eagle's medium, BioWhittaker) containing 10% heat-inactivated fetal bovine serum (HI-FBS, BioWhittaker), 2 mM L-glutamine (BioWhittaker), 1 μM dexamethason (Sigma), 10 μg/mL insulin, 5.5 μg/mL transferrin, 6.7 ng/mL selenium-X (ITS mix, Life Technology), 100 U/mL penicillin, 100 μg/mL streptomycin (penicillin/streptomycin mix, BioWhittaker) at a density of approximately 3*10^5 ^cells/cm^2 ^in Primaria 6-well plates (BD Falcon). Clonal derivatives were obtained by limiting dilution. The selection procedure used and the functionality of the clonal derivatives are described elsewhere [14]. Near-confluent cultures were detached by 5 min incubation with 0.25% trypsin/0.03% EDTA (BioWhittaker) and split at 1:4 ratios. The number of population doublings (PD) was calculated as PD = log (N_f_/N_i_)/log 2, in which N_f _is the final number of cells harvested and N_i _is the number of cells initially seeded. No corrections were made for cells that did not re-attach after passaging, since their proportion was negligible. The period in which PD number progressed linear with culture time was used to calculate the PD time (T_PD_).

Mature primary human hepatocytes were isolated from seven patients undergoing partial hepatectomy, because of metastatic carcinoma. The tumour free liver tissue used in each case for the hepatocyte isolation ranged between 2 to 10 grams. The procedure was approved by the Medical Ethical Committee of the Academic Medical Center subject to informed patient consent. The hepatocyte isolation method was adapted from the protocol described by Seglen [15] as previously described [14].

NKNT-3 cells were kindly donated by Prof. I. Fox, University of Nebraska, USA. The NKNT-3 cells were cultured on Primaria 6-well culture plates (BD Falcon) and in 75 cm2 culture flasks using CS-C complete serum free medium (Cell Systems Corporation) with 0.2 mg/ml hygromycin B (Invitrogen) and 1 U/ml penicillin/streptomycin (BioWhittaker). Cultures were passaged with a split ratio of 1:5 according to instructions for CS-C medium. Cre-mediated recombination to revert immortalization of NKNT-3 cells [4] was carried out by transduction of the adenoviral vector AxCANCre (Riken DNA Bank (Tsukuba Life Science Center, Japan) as described previously [5]. We analysed both reverted, hence transduced with AxCANCre and selected with G418, and unreverted *i.e*. untreated cells. HepG2 cells were obtained from ATCC (HB-8065) and cultured in Primaria tissue culture flasks in DMEM culture medium as described above for HFLCs.

All cultures were maintained at 37°C in a humidified atmosphere (95% air, 5% CO2) and the medium was changed every 2-3 days.

### Introduction of hTERT and Green Fluorescent Protein genes

The cDNA of the human telomerase reverse transcriptase (hTERT) gene, kindly provided by R.L. Beijersbergen, Netherlands Cancer Institute, the Netherlands, was introduced in the cells by lentiviral transduction. The lentiviral vector backbone was described as LTRCMVR2 by Marcusic et al. [16]. The lentiviral vector contained a cytomegalovirus promoter controlling the expression of a reverse tetracycline (Tet) responsive transcriptional activator and a Tet responsive element controlling the expression of the hTERT gene. In this system hTERT transcription can be increased by adding 1 μg/mL doxycyclin to the medium.

The lentivirus was produced as previously described [17]. In brief, HEK293T cells were transiently transfected by calcium phosphate precipitation with a third generation lentiviral vector system. Virus containing supernatant was collected at 24 and 48 hours following transfection, filtered through 0.45 μm Millipore filters, then 100-fold concentrated by centrifugation and added to the culture medium of the HFLCs in 24-well culture plates in a 1:50 (lentivirus preparation:culture medium) ratio. The hTERT lentivirus was introduced in three independent HFLC cultures and in five clonal derivatives, *i.e*. cBAL08, cBAL09, cBAL20, cBAL21 and cBAL29. Cells were passaged twice before integration of the lentiviral vector was confirmed by PCR using genomic DNA of transduced cells as template.

In the transplantation experiment cBAL111 cells were marked with Green Fluorescent Protein (GFP) by transduction using lentiviral construct pRRLcpptPGKGFPpreSsin [17] carrying the GFP gene under control of a phosphoglycerate kinase promoter. By fluorescence-activated cell sorting it was demonstrated that ≥ 95% of the transduced cells were GFP positive. There were at least five passages between transduction with GFP and transplantation of the cells. The GFP positivity, ratio as well as expression level, of the cells remained unchanged during passaging.

### Flow cytometry

The cBAL111 cells, cultured for 15 days, were analyzed for progenitor cell markers by flow cytometry. For comparison HepG2 cells were included in the experiment. The cells were detached using accutase (Innovative Cell Technologies, Inc., USA), washed twice in DMEM culture medium, resuspended in 100 μl DMEM and incubated for 30 minutes on ice in the dark with 10 μl of the following antibodies: 1. a combination of mouse-anti human CD34-FITC and mouse-anti human CD326 (EpCAM)-APC (both Miltenyi Biotech Inc.); 2. a combination of mouse-anti human CD146-FITC (Miltenyi Biotech Inc., USA) and mouse-anti human CD326 (EpCAM)-APC; 3. mouse-anti human CD133 (Miltenyi Biotech Inc., USA). The cells incubated with the CD133 antibodies were washed in culture medium and subsequently incubated with the secondary antibody goat-anti mouse IgG-Alexa fluor 488 (Molecular Probes Invitrogen, USA). As negative controls, cells were incubated with either a combination of mouse IgG2a-APC and mouse IgG1-FITC (isotype controls) (both eBioscience, USA) or with goat-anti mouse IgG-Alexa fluor 488. The cells were analyzed by flow cytometry using BD FACScalibur (BD Biosciences, USA) and data were analyzed with WinMDI 2.8 software.

### Hepatocyte function tests

Hepatocyte function tests were performed at confluence in 6-well plates. After washing the cells twice with phosphate buffered saline (PBS, NBPI International) culture medium was replaced by 2.5 mL of test medium (William's E medium with 4% HI-FBS, 2 mM L-glutamine, 1 μM dexamethason, 20 mU/mL insulin (Novo Nordisk), 2 mM ornithine (Sigma-Aldrich), 100 U/mL penicillin, 100 μg/mL streptomycin, 0.5 mM NH4Cl, 2.75 mM D-galactose (Sigma), 90 μM lidocaine HCl (Sigma-Aldrich). Medium samples were taken after 0 and 72 hours of incubation. The cells were then washed twice with PBS and stored at -20°C for protein determination.

### Biochemical assays

Urea concentrations were determined using the blood urea nitrogen test (Sigma Chemical Co). Albumin concentrations were determined via enzyme linked immunosorbent assays using cross-absorbed goat-anti-human albumin antibodies (Bethyl). Lidocaine concentrations were measured by fluorescence polarization immunoassay using a TDxFLx analyzer (Abbot Laboratories). Galactose concentrations were determined by the absorbance of nicotinamide-adenine dinucleotide (NADH) at 340 nm after enzymatic reaction with galactose dehydrogenase (Roche). Total protein/well was quantified by spectrometry using Coomassie blue (Bio-Rad). Production rates were established by calculating the changes in concentration during time and corrected for protein content.

### RT-PCR

RNA was isolated from the cell lines by using TRIzol (Boehringer Mannheim). As a reference, human liver samples were included in the analyses. First strand cDNA was generated from 500 ng of total RNA using 20 pmol of gene-specific RT primers specific for the mRNA of Albumin, α-1-Antitrypsin (AAT), Transferrin, Hepatocyte Nuclear Factor 4 α (HNF4α), Alpha-fetoprotein (AFP), π class Glutathione S transferase (GST π) and hTERT in combination with 5 pmol of RT primer for 18S ribosomal RNA and 134 units Superscript III (Invitrogen). Real-time reverse transcription PCR (RT-PCR) using SYBR green I (Roche) was performed as described previously [18]. The sequences of the RT and PCR primers and PCR conditions are given in Table 1.

**Table 1:** Primers and conditions used in RT-PCR analysis

**Gene**	**Sense primer 5' → 3'**	**Antisense primer 5' → 3'**	**Application**	**Size amplicon (bp)**	**PCR conditions**	
					
					**Dilution template**	**Annealing temp. (°C)**
***18S rRNA***	NA	CGAACCTCCGACTTTCGTTT	RT		NA	

***AAT***	NA	GGGGGATAGACATGGGTATGG	RT		NA	

***AFP***	NA	CGTTTTGTCTTCTCTTCCCC	RT		NA	

***Albumin***	NA	ACTTCCAGAGCTGAAAAGCATGGTC	RT		NA	

***GSTπ***	NA	AGCAGGTCCAGCAGGTTG	RT		NA	

***HNF4α***	NA	CACTCCAACCCCGCCCCTC	RT		NA	

***hTERT***	NA	CAGAGCAGCGTGGAGAGGATG	RT		NA	

***Transferrin***	NA	CCAGACCACACTTGCCCGCTATG	RT		NA	

***18S rRNA***	TTCGGAACTGAGGCCATGAT	CGAACCTCCGACTTTCGTTT	PCR	151	1000×	68 → 63

***AAT***	ACAGAAGGTCTGCCAGCTTC	GATGGTCAGCACAGCCTTAT	PCR	181	-	68 → 63

***AFP***	TKCCAACAGGAGGCYATGC	CCCAAAGCAKCACGAGTTTT	PCR	306	-	62 → 55

***Albumin***	TGAGCAGCTTGGAGAGTACA	GTTCAGGACCACGGATAGAT	PCR	189	-	68 → 63

***GSTπ***	GCCAGAGCTGGAAGGAGG	TTCTGGGACAGCAGGGTC	PCR	333	10×	70 → 63

***HNF4α***	CCGGGTGTCCATACGCATCCT	CAGGTTGTCAATCTTGGCC	PCR	321	-	68 → 63

***hTERT***	CGTACTGCGTGCGTCGGTAT	GGTGGCACATGAAGCGTAGG	PCR	233	-	68 → 63

***Transferrin***	GAAGGACCTGCTGTTTAAGG	CTCCATCCAAGCTCATGGC	PCR	310	-	68 → 63

Sequences of the primers used in Reverse Transcriptase (RT) reaction or during real-time PCR reaction (PCR) and the conditions used in the real-time PCR reactions. NA = not applicable.

Starting levels of mRNA, except for hTERT, were calculated by analyzing linear regression on the Log (fluorescence) per cycle number data using LinRegPCR software [19]. Starting levels of hTERT mRNA were calculated by standard curve analysis, using serial dilutions of hTERT containing plasmid ranging from 10^2 ^to 10^9 ^copies/reaction and LightCycler software (Roche). The mRNA starting levels of Albumin, AAT, Transferrin, AFP, GST π, and hTERT were normalised for the starting levels of 18S ribosomal RNA. Normalised mRNA levels, except for hTERT mRNA levels, are expressed as a percentage of the mean mRNA starting levels of the two liver samples normalised for 18S ribosomal RNA starting levels.

### Immunocytochemistry

For the detection of glutamine synthetase (GS), cBAL111 cells were cultured on 8-wells culture-slides (BD Falcon) for two days. Then the cells were washed twice with PBS and fixed by a 10-minutes incubation with ice-cold methanol-aceton-water mixture (2:2:1). Cells were incubated with 70% ethanol for 5 minutes, washed with PBS and subsequently incubated overnight with monoclonal GS antibody (Transduction Laboratories, Lexington, KY, G45020) diluted 1:1000. Antibody binding was visualized with the indirect unlabelled antibody peroxidase anti-peroxidase (PAP) method [20].

For the detection of albumin, cytokeratin (CK) 18 and CK19, cBAL111 cells were seeded on Immunoslides (ICN, Aurora, Ohio, USA) and cultured for two and 15 days. Cells were washed once in PBS with 0.1% Tween-20 and fixated in 4% paraformaldehyde in PBS for 15 minutes at room temperature. Cells were washed as before and incubated with a blocking buffer (3% BSA, 0,2% Fish gelatin (Sigma), 2%FCS) for one hour at room temperature. After a further washing step, the cells were incubated with the primary antibody for one hour at room temperature. As primary antibodies we used anti-albumin antibody (Sigma), mouse-anti-human CK18 (sc-6259, Santa Cruz) and mouse-anti-human CK19 (Santa Cruz) for detection of albumin, CK18 and CK19, respectively. Cells were washed again as before and incubated with 28 μg/mL Cy2 conjugated goat-anti-mouse IgG and 1 μg/mL tetramethylrhodamine isothiocyanate (TRITC) conjugated phalloidin for 1 hour in a humidified chamber at room temperature. Forty minutes before the end of this incubation period, 20 ng/mL Diamidinophenylindoldiacetate (DAPI) was added. Cells were washed again as before and embedded in Polymount (Polyscience, Washington, USA) and covered with a coverslip. Slides were analysed using an Axiovert 200 fluorescence microscope.

### Soft Agar assay

Cells were added to 0.35% low-melting-temperature agarose (Seaplaque) containing DMEM culture medium as described above and transferred at a density of 5000 cells/well to 6-well plates previously lined with 0.5% agar DMEM culture medium. After 15 days, the colonies were stained with 0.005% Crystal violet and counted.

### Transplantation

The cBAL111 cells overexpressing GFP were transplanted into four 6 week old Rag2-/-γc-/- mice [21]. The mice were anesthetized with an intraperitoneal injection of FFM mixture (2.5 mg Fluanisone/0.105 mg Fentanyl citrate/0.625 mg Midazolam HCl/kg in H_2_O, 7 mL/kg). One million GFP-marked cBAL111 cells suspended in 100 μL HBSS were injected into the inferior tip of the spleen as described [22]. At both nine and 34 days after transplantation, two mice were sacrificed. The livers and spleens were harvested after *in vivo *fixation. For *in vivo *fixation, mice were anesthetized as described above and 25 mL PBS was flushed trough the circulation, followed by 25 mL 2% paraformaldehyde (PFA) in PBS. Subsequently, liver and spleen were harvested and cut into pieces of approximately 0.2 cm^3^. The tissue pieces were incubated in 4% PFA in PBS for four hours, followed by an overnight incubation in 30% sucrose solution. Tissues were snap frozen in liquid nitrogen and stored at -80°C.

### Immunohistochemistry

Cryosections were 6 μm thick and were mounted on poly-L-lysine coated slides. Sections were incubated in Teng-T (10 mM Tris, 5 mM EDTA, 0.15 M NaCl, 0.25% gelatin and 0.05% Tween-20, pH 8.0) for 30 minutes before incubation with primary antibodies. Human mitochondria were visualized with a mouse-anti-human mitochondria antibody (Chemicon International) in a 150-fold dilution; vimentin was visualized with a mouse-anti-vimentin antibody, clone 9 (Boehringer Mannheim) in a 1000-fold dilution; carbamoylphosphate synthetase (CPS) was visualized with rabbit-anti-CPS antibody in a 1500-fold dilution and glutamine synthetase (GS) was visualized with monoclonal GS antibody (Transduction Laboratories) in a 500-fold dilution, human albumin was visualized with goat-anti-human albumin antibody in a 1:200 fold dilution. As a secondary antibody Alexa594 conjugated goat-anti-mouse IgG (Molecular Probes) was used in a 1000-fold dilution for the detection of vimentin and human mitochondria and in a 250-fold dilution for detection of GS. CPS antibodies were detected with Alexa594 conjugated goat-anti-rabbit IgG (Molecular Probes) in a 250-fold dilution. Goat-anti-human albumin immunoglobulins were detected using rabbit-anti-goat IgG conjugated with Alexa 594 (Molecular Probes). Slides were mounted in Vectashield containing 1 μg/mL 4,6-diaminidino-2-phenylindole (DAPI) to counterstain DNA.

### FISH analysis

Fluorescent in situ hybridization (FISH) analysis was performed on 6 μm sections of paraffin embedded liver tissue as described before [23]. Briefly, sections were treated to remove paraffin and the sections were denatured in 70% formamide for 2.5 minutes. 150 ng Biotin 11-dUTP labeled human genomic DNA, 200 ng digoxigenin 11-dUTP labeled murine genomic DNA and 5 μg salmon sperm DNA were denatured together and hybridized overnight at 37°C with the sections. After washing the slides, human DNA was visualized with FITC anti-avidin followed by biotinylated anti-avidin antibodies (Vector laboratories), whilst mouse genomic DNA was visualized with sheep rhodamine anti-digoxigenin followed by Texas red anti-sheep antibodies (Vector laboratories). Slides were mounted in Vectashield (Vector laboratories) containing 1 μg/mL DAPI to counterstain DNA.

### Statistical analysis

Student's t tests were used to determine statistical differences. Significance was reached if *P *< 0.05. SPSS 12.0.1 (SPSS Inc., Chicago, IL, USA) was used for statistical analysis. Average values (± standard error) are reported.

## Results

### Human TERT introduction in HFLCs and clonal derivatives

The maximum number of PDs between the HFLC preparations and clonal derivatives were different. Expression of hTERT was detected in some of the clonal derivatives analysed, however, all HFLCs and clonal derivatives eventually entered a state of terminal growth arrest (Table 2).

**Table 2:** The life span and the hTERT mRNA levels of three different HFLC isolates, clonal derivatives and cBAL111

**Cell source**	**Maximal PDs**	**hTERT mRNA copies/18S rRNA copies**
HFLCs, 16 weeks	57.6 ± 10.2	Undetectable (n = 4)

cBAL08	61	2.7 *10^4 ^(n = 1)

cBAL09	30	Not determined

cBAL20	38	Undetectable (n = 1)

cBAL21	31	Undetectable (n = 1)

cBAL29	42	2.4 *10^5 ^± 1.9 *10^5 ^(n = 3)

cBAL111	Immortal	2.6 *10^9 ^± 3.2 *10^8 ^(n = 4)

Life span is indicated as the maximum number of population doublings (PDs).

After lentiviral introduction of the hTERT gene, the presence of the hTERT cDNA was confirmed in HFLCs and clonal derivatives by PCR on genomic DNA (results not shown). However, the HFLC cultures did not overcome the terminal growth arrest after the introduction of the hTERT gene; only one of the HFLC cultures' lifespan was extended by 30%. In addition, only one of the clonal derivatives was able to overcome the terminal growth arrest after the introduction of hTERT. This clone, cBAL08, previously showed the longest life span of 61 PDs and a relatively high endogenous hTERT expression (Table 2). Because the transduced cell line was capable of more than 120 PDs, which is twice the life span of the parental cell line cBAL08, and still did not show any sign of growth arrest, we considered this cell line to be immortalized and named it cBAL111. The hTERT mRNA levels of cBAL111 were 1 × 10^5^-fold higher as compared to its parental cell line cBAL08 (Table 2).

### Characterisation of cBAL111 *in vitro*

**Figure 1:**
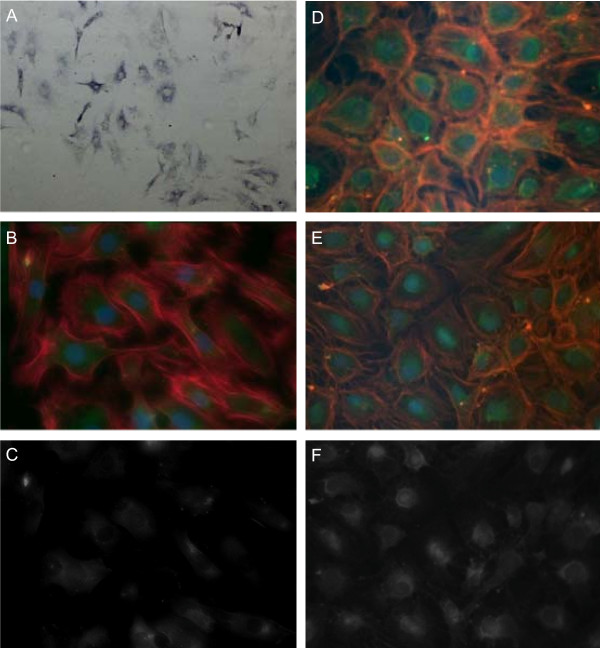
**Immunostainings of cBAL111 cells *in vitro***. The cells were stained using antibodies against GS (A) (blue, 40× magnification, 2 days in culture), albumin (B, C) (green, 400× magnification, 2 days in culture) and CK19 (D) or CK18 (E, F) (both green, 400× magnification, 15 days in culture). In the CK18, CK19 and albumin staining, cells were further visualized by phalloidin, binding to actin (red), and DAPI, binding to the nuclei (blue) (B, D, E). Corresponding single stains of albumin and CK18 are shown in C and F.

The organisation of the cBAL111 cell layer changed during culturing; the cells displayed a cubic shape at day 15 (Fig. 1C and 1D) instead of the more spindle shape at day 2 (Fig. 1A and 1B). The cBAL111 cultures were 100% positive for the hepatocyte markers GS, CK 18 and albumin as well as the cholangiocyte marker CK 19 at day 2 and day 15 after seeding (GS and albumin are shown at day 2 and CK18 and CK19 are shown at day 15 in Fig. 1). The CK18 staining was predominantly around the nucleus reaching into the cytoplasm, which is similar to the CK18 staining of primary human hepatocytes that are dedifferentiating *in vitro *[24]. These data suggest that under these conditions the cBAL111 cells resemble cells with progenitor characteristics rather than fully differentiated hepatocytes.

**Figure 2:**
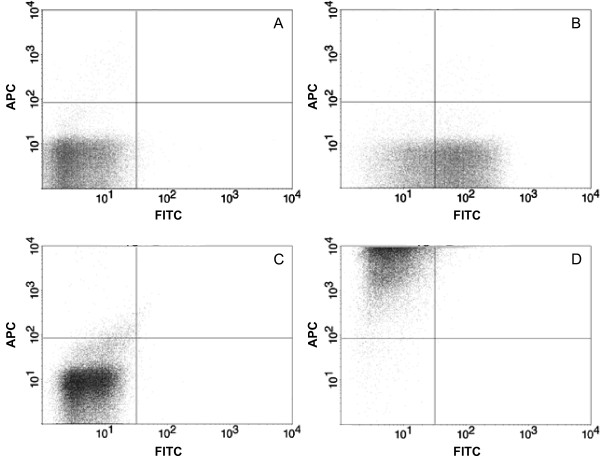
**Dotplots of flow cytometry analysis of CD146 and CD326 expression of cBAL111 cells (A, B) and HepG2 cells (C, D)**. (A, C) Negative isotype controls, (B, D) cells labeled with anti-CD146-FITC and anti-CD326-APC.

The 15 day-cultured cBAL111 cells and, for comparison, hepatoblastoma derived HepG2 cells were characterized for three progenitor cell markers by flow cytometry, *i.e*. CD146, a marker for various cells including mesenchymal progenitor cells and hepatocytes during liver regeneration [25,26], CD326 (EpCAM), an epithelial marker upregulated in gastrointestinal carcinomas [27] and CD133, a marker for progenitor cells of various origins, like endothelial cells, haematopoietic cells and neural cells [28]. The cBAL111 cells were positive for CD146, but negative for CD326 (EpCAM) (Fig. 2A and 2B) and CD133 (data not shown). HepG2 cells were also negative for CD133 (data not shown), but, in contrast to the cBAL111 cells, positive for CD326 and negative for CD146 (Fig. 2C and 2D).

Because the immunostainings suggested a transition in the organisation of the cell layer of cBAL111 cells, we tested their in vitro functionality at 2 days of culture, when the cells displayed a spindle like morphology and the culture was not confluent and at day 15, when cells were more cubic and confluence was reached. There was a trend of increased expression of markers for mature hepatocytes (production of albumin and urea, galactose elimination and transcript levels of transferrin, AAT and HNFα) with culture time and decreased expression of markers for immature hepatocytes (GSTπ and AFP) (Table 3). The *in vitro *functionality of cBAL08 after reaching confluence was comparable with that of cBAL111, particularly at day 15; no significant difference was detected between cBAL08 and cBAL111 at day 15 for urea and albumin production and the mRNA levels of Albumin, AAT, Transferrin, GSTπ and AFP differed less than 2.5-fold (Table 3). So, the immortalization of cBAL08 maintained the investigated hepatic functions. Further extension of the culture period did not clearly increase hepatic functionality (results not shown).

**Table 3:** Hepatic functions of different hepatic cell lines and primary mature human hepatocytes (Mat Hep)

	**Hepatic function (n = 9)**			**mRNA levels (n = 3)** **Mature hepatic marker**				**mRNA levels (n = 3)** **Immature hepatic marker**	
	
**Cell**	**Albumin production** **(ng/h/mg protein)**	**Urea production** **(nmol/h/mg protein)**	**Galactose elimination** **(μmol/h/mg protein)**	**Albumin**	**Transferrin**	**AAT**	**HNF4**	**GSTπ**	**AFP**
Mat Hep	37.7 ± 7.7	91.5 ± 33.7	0.58 ± 0.44	130	245	121	141	535	ND

cBAL08	0.3 ± 0.4	4.0 ± 1.6	ND	0.02 ± 0.01	0.20 ± 0.14	0.32 ± 0.14	ND	1000 ± 565	78 ± 61

cBAL111 day 2	Undetectable	Undetectable	0.04 ± 0.02	0.02 ± 0.01	0.11 ± 0.05	0.02 ± 0.01	1.3 ± 1.3	1944 ± 1010	122 ± 11

cBAL111 day 15	0.7 ± 0.8	8.0 ± 6.6	0.11 ± 0.03*	0.02 ± 0.01	0.20 ± 0.06	0.05 ± 0.01	17.2 ± 5.6*	1374 ± 671	114 ± 10

HepG2	2.8 ± 0.3	4.7 ± 0.2	ND	63 ± 6	896 ± 110	199 ± 56	ND	Undetectable	93353 ± 13228

NKNT-3 reverted	Undetectable	4.9 ± 9.4	ND	0.24 ± 0.22	0.16 ± 0.12	0.92 ± 1.52	ND	952 ± 1164	117 ± 166

NKNT-3 unreverted	Undetectable	2.9 ± 5.2	ND	0.42 ± 0.66	0.20 ± 0.13	0.26 ± 0.34	ND	4427 ± 5366	65 ± 79

MessengerRNA levels are expressed as a percentage of the mean mRNA levels of the two liver samples. ND = not determined

* cBAL111 day 2 *versus *cBAL111 day 15; P < 0.05

To investigate the putative tumorigenicity of cBAL111, the cells were seeded in soft agar. The cBAL111 cells were not able to form colonies in soft agar, where HepG2 as positive control, formed 61 ± 21 colonies from 5000 cells. The inability of cBAL111 cells to grow in an anchorage independent way, is an indication that cBAL111 cells are not tumorigenic.

### Comparison of cBAL111 with other hepatic cell lines and mature hepatocytes

The novel *in vitro *immortalized fetal hepatocyte cell line cBAL111 was subsequently compared with HepG2 cells, the conditionally immortalized hepatocyte cell line NKNT-3 and primary (mature) human hepatocytes two days after seeding. The primary human hepatocytes did not exhibit any decline in the tested functions within two days (results not shown). HepG2, cBAL111 at day 15 and NKNT-3 cells, both un-reverted and reverted for immortalization, synthesized urea, at a level 11-32 fold lower than mature hepatocytes (Table 3). The HepG2 cells and to a lesser extent the cBAL111 cells produced albumin in contrast to NKNT-3 cells. The galactose elimination of the cBAL111 cells at day 15 reached to 19% of the levels of the mature hepatocytes. The cBAL111 cells, like fetal hepatocytes, did not eliminate lidocaine, a marker for cytochrome P450 1A2 and 3A4 activity, whereas the mature hepatocytes eliminated 9.8 ± 9.3 nmol/h/mg protein.

In cBAL111 and NKNT-3 cells, the mRNA levels of albumin, AAT and transferrin, (markers of hepatocyte differentiation) were less than 1% of the corresponding levels in human liver. However, the transcript levels of HNF4α, a master regulator of liver development, increased up to 17% of the human liver levels in the cBAL111 cells at day 15. HepG2 cells showed mRNA levels for albumin, AAT and transferrin that were comparable to human liver. As markers associated with immature hepatocytes, AFP mRNA levels of cBAL111 and NKNT-3 cells were comparable to the *in vivo *level, while the GST π mRNA levels were 10-44 times higher. In HepG2 cells, mRNA levels for AFP were 1000-fold higher than the *in vivo *levels and GST π levels were undetectable. In summary cBAL111 at day 15 was positive for all tested hepatocyte parameters, whereas HepG2 and NKNT-3 cells both lacked one function.

### cBAL111 cells differentiate into functional hepatocytes in murine liver

**Figure 3:**
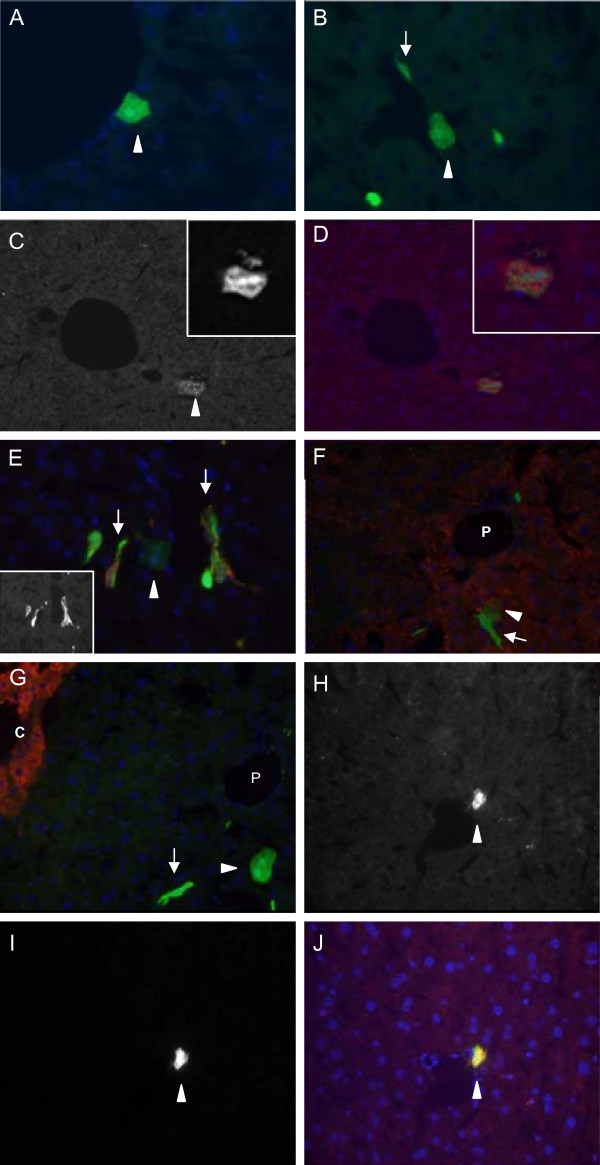
**Immunofluorescence of mouse livers (40× magnification), harvested 9 days after transplantation of GFP marked cBAL111 cells (green)**. Nuclei were visualized by DAPI staining (blue) (A-F, I). The majority of cells exhibited an elongated morphology (arrows); a small number of cells adapted hepatocyte morphology (arrowheads, A and B). Human origin of the cells was confirmed using an antibody against human mitochondria (red) (C and D); Human mitochondria fluorescence (C), GFP fluorescence (C insert) and merge of human mitochondria and GFP fluorescence (D, insert shows higher magnification). With hepatic differentiation, the cells lost the expression of vimentin, a marker of undifferentiated mesenchymal cells (red; insert shows single stain) (E). Hepatocytes originating from cBAL111 are undistinguishable from the surrounding murine hepatocytes in CPS expression (red) (F). No cells originating from cBAL111 were found in pericentral regions; cBAL111 *in vivo *did not express GS (G). Transplanted cBAL111 cells with hepatocyte morphology showed co-localization of GFP and human albumin (red) (H-J); albumin fluorescence (H), GFP fluorescence (I) and merge of albumin and GFP fluorescence (J). P, portal vein; C, central vein.

To determine whether cBAL111 cells have the potential for hepatic differentiation, the cells were marked with GFP by lentiviral transduction and transplanted in the spleen of 4 immunodeficient mice. Nine and 34 days after transplantation, GFP expressing cells were detected in the murine spleen (results not shown) and liver (Fig. 3A and 3B). The presence of GFP positive cells in the murine livers was relatively low (~1%), since no liver repopulation model was applied, and remained stable between nine and 34 days. The cells appeared as single cells and no cell clusters were detected, which again suggested a lack of tumorigenicity.

The majority of these cells exhibited an elongated morphology, however a small number of cells (~1%) had the morphological characteristics of hepatocytes, given their cuboid appearance. No differences were observed between the livers harvested at nine and 34 days after transplantation. The GFP positive cells were confirmed to be from human origin by immunohistochemistry using a human specific antibody binding to mitochondria (Fig. 3C and 3D). Immunohistochemistry using an antibody against vimentin, a marker for dedifferentiated and mesenchymal cells [29], indicated a high expression in cBAL111 *in vitro *(data not shown) and in all the elongated cBAL111 cells found in the mouse liver, whereas the cBAL111 cells with cuboid appearance did not or hardly expressed vimentin (Fig. 3E). Furthermore these GFP positive hepatocyte-looking cells with cuboid morphology were indistinguishable from the surrounding mice hepatocytes with regards to CPS expression, which is expressed periportally [30] (Fig. 3F). In contrast, elongated cBAL111 cells did not express CPS. GS expression, which is present *in vitro *(Fig. 1), was absent in the transplanted cells in the periportal areas (Fig. 3G). No GFP positive cells were detected in pericentral areas, the site of GS expression in normal liver. A possible explanation for this is that the cells may have entered the liver via the portal vein and engrafted before reaching the pericentral area. Finally, the cuboid GFP positive cells stained positive for human albumin (Fig. 3H-J), whereas the elongated GFP positive cells were negative (not shown).

**Figure 4:**
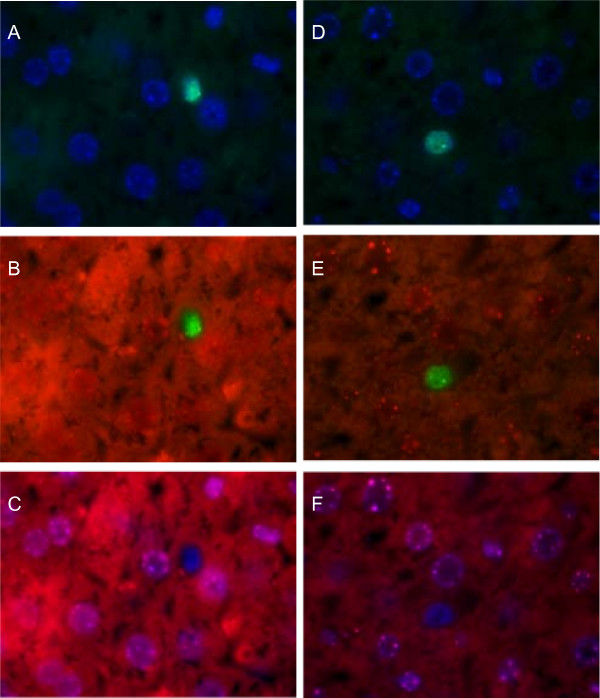
**FISH analysis (40× magnification) of mouse livers after transplantation of GFP marked cBAL111 cells**. The probe hybridizing with human DNA was visualized using a FITC labeled antibody (A, B, D and E), showing green nuclei, the probe hybridizing with murine DNA was visualized using a Texas Red labeled antibody (red nuclear staining on top of cytoplasmic background autofluorescence, B, C, E and F). All nuclei were counterstained using DAPI (blue A, C, D and F). Picture sets A-C and D-F both show a human cell that is negative for the murine DNA.

We then tested whether the GFP positive hepatocytes were the result of fusion between human cBAL111 cells and murine hepatocytes [31], by using FISH analysis. The results showed that nuclei reacting to the human probe (Fig. 4A and 4D) were negative for the murine probe (Fig. 4C and 4F). From the shape of the nuclei and the localization of cells, we concluded that these were cBAL111 derived hepatocytes.

## Discussion

In this report we present a novel, clonal, immortalized human fetal liver cell line, cBAL111, that displays hepatocyte-specific functions. *In vitro*, the cBAL111 cell line produced albumin and urea and expressed hepatocyte-specific genes. However, the levels of the hepatic functions showed considerable variation compared with primary mature hepatocytes. Moreover, the cBAL111 cells expressed immature markers CD146, cytokeratin 19 and glutathione S transferase π, but did not show any evidence of growth in soft agar. *In vivo*, when cBAL111 was transplanted in immunodeficient mice, the cell line showed the potential to fully differentiate into hepatocytes and adapt the zonal expression pattern of CPS.

Human fetal liver cells have been immortalised by telomerase activation before [12], but this is the first report in which this immortalisation strategy was combined with a cloning procedure. In contrast to Wege *et al*. we could not immortalise unselected HFLC preparations [12], although we were able to extend the life span of the cells by about 30%. In our study we selected cells with cuboid morphology from the initial cell preparation and of these selected cells only the cell line with the highest proliferation capacity, cBAL08, became immortal by overexpression of hTERT. The reduced ability of immortalization in the present study may be the result of differences in transduction levels observed in the two studies. Wege *et al *used a constitutively active Moloney murine leukemia promoter to drive hTERT expression [12]; we used a tetracycline inducible expression system, stimulated with 1 μM doxycyclin, to drive hTERT expression. Because Moloney murine leukaemia vectors are prone to transduce rapidly dividing cells, this might have resulted in the selection of cells prone to immortalization.

Our results are in line with the conclusion of Wege *et al *that telomerase-induced immortalization of HFLCs does not affect their differentiation potential [12]. After immortalisation, cBAL111 was capable of reaching urea and albumin production rates and the hepatocyte specific mRNA levels comparable to the levels of cBAL08. However, cBAL111 needs prolonged cultivation time to produce albumin and urea. In addition, galactose elimination increased after prolonged culturing. This may be related to the increased transcript levels of HNF4α, a key regulator of hepatocyte development and function [32].

Furthermore, we found no indications that cBAL111 was malignantly transformed; cBAL111 was not able to form colonies in soft agar and no tumours were found in immunodeficient mice 34 days after transplantation. We conclude that telomerase reconstitution can immortalize fetal human hepatocytes without loss of function and without indications of malignant transformation.

To our knowledge this is the first time that a hTERT-immortalized human fetal liver cell line was evaluated for hepatic function *in vitro*. The clonal origin of the cell line is most important for preservation of the phenotype during long-term culturing. The stability in phenotype was previously shown for cBAL08 [14]. This is in contrast to cultures of hepatocyte isolates from mature or fetal origin, either immortalized or not.

In addition to the description of cBAL111, we compared its functionality with that of mature human hepatocytes kept under the same culture conditions. It is rather surprising that such comparison is rarely seen in similar studies describing hepatic cell lines. Mature hepatocytes are currently the only cells that meet the criteria for *in vitro *applications and therefore are the gold standard when evaluating alternative hepatic cell lines. The performance of our novel hepatocyte line cBAL111 was also compared with two well-known and widely used hepatic cell lines, NKNT-3 [4] and HepG2 [3]. Admittedly all three cell lines, performed considerably less well compared with primary mature human hepatocytes in all the liver parameters tested in this study. NKNT-3 cell did not produce detectable levels of albumin; HepG2 cells lacked detectable GST π expression. In concordance with the low level of hepatic differentiation, relatively high mRNA levels of AFP and particularly GST π were observed in cBAL111. Moreover the cBAL111 cells expressed CK19, a marker for cholangiocytes, and in addition CK18, a marker for hepatocytes, in a pattern characteristic of de-differentiated human hepatocytes. Finally, the cBAL111 cells expressed CD146, a marker of various cells, including mesenchymal progenitor cells [25], Therefore it can be concluded that cBAL111 cells are not fully differentiated *in vitro *into mature hepatocytes, but rather should be regarded as a progenitor liver cell line that has the full potential to differentiate into hepatocytes. These cells may derive from mesodermal cells, as also suggested for multipotent progenitor cells previously isolated from human fetal liver [33].

Interestingly, HepG2 and cBAL111 showed contrasting expression patterns of CD146 and CD326. Both membrane glycoproteins are expressed on progenitor cells. However, CD326 is heavily upregulated in premalignant hepatic tissues and hepatocellular carcinomas and a marker for tumor-initiating stem cells, in contrast to CD146 [34,27,35]. Expression of CD146 is low in mature hepatocytes, but is upregulated in the termination phase of liver regeneration and in primary hepatocytes in the presence of transforming growth factor (TGF)-β1. These observations underline the non-transformed phenotype of cBAL111 cells in contrast to HepG2 cells.

The liver provides an optimal environment for hepatic differentiation of cBAL111. When cBAL111 cells were labelled with GFP and transplanted into the spleen of immunodeficient mice the cells migrated to the liver. This was already shown with human fetal hepatocytes, both immortalized and freshly isolated [36,12]. However, in this study we showed that following engraftment a number of these cells went to become hepatocytes morphologically indistinguishable from murine hepatocytes. These cells had very low or no expression of vimentin, a marker for mesenchymal cells and for the undifferentiated cBAL111 cells. In addition, the cuboid cells expressed human albumin and adapted the zonal expression pattern of CPS and GS characteristic for the surrounding cells [30]. With FISH analysis, we excluded the possibility that these GFP positive hepatocytes were the result of fusion between GFP labelled cBAL111 cells and murine hepatocytes. Fusion between host liver cells and transplanted stem cells, specifically haematopoietic stem cells, has been widely reported to account for high rates of transdifferentiation [37,31]. Our experiments confirm that the cBAL111 line is able to differentiate into hepatocytes when the right differentiation stimuli are present.

However, the fact that a significant number of the transplanted cells did not adapt the hepatocyte morphology and expressed high levels of vimentin, but no human albumin, suggests that either not all cBAL111 cells were equally sensitive to differentiation stimuli, despite the clonal origin of the cells, or that not all cells were exposed to the same levels of differentiation stimuli due to micro-environmental variations. This requires further investigation.

Very recently we have tested the cBAL111 cells in the AMC-bioartificial liver (AMC-BAL), which is a bioreactor, more suitable for hepatocyte culturing than monolayer culturing [38,39]. We showed that the cBAL111 cells eliminated ammonia and galactose at a rate up to 49% and 90% of that of primary porcine hepatocytes in the AMC-BAL, respectively. Other functions, like albumin production and lidocaine elimination only reached 6% and 0.1% of the levels of primary porcine hepatocytes in the AMC-BAL. This further underlines that the cBAL111 cells are indeed hepatocyte-like cells which probably need further stimulation of hepatic differentiation for *in vitro *applications.

## Conclusion

The development of a cell line that combines both *in vitro *hepatic function and proliferation capacity is important for large-scale applications that depend on *in vitro *hepatic functionality. In this study we present evidence of a novel cell line cBAL111, which is a telomerase immortalized fetal human hepatocyte cell line capable to differentiate into mature hepatocytes *in vivo*. The potential of this novel cell line merits further investigation. The challenge is to define the best possible experimental conditions *in vitro *to mimic as closely as possible the differentiation stimuli present *in vivo *aiming to achieve a high degree of differentiation into mature hepatocytes *in vitro*.

## Authors' contributions

TD carried out the immortalization and *in vitro *characterization of cBAL111 and drafted the manuscript. NPvT carried out the transplantations. AAC participated in the molecular biological studies. LtB participated in the molecular biological studiesRS carried out immunostainings. CP carried out the FISH analysis. JP coordinated the FISH analysis and helped to draft the manuscript. IMS coordinated the immunostainings. RAFMC conceived of the study, participated in the design of the study and coordination and helped to draft the manuscript. RPJOE participated in the design of the study and coordination and helped to draft the manuscript. JS participated in the design of the study and coordinated the transplantations. RH conceived of the study, participated in the design of the study, coordination and molecular biological studies and helped to draft the manuscript.

All authors read and approved the final manuscript.
